# CHOP Pro-Apoptotic Transcriptional Program in Response to ER Stress Is Hacked by Zika Virus

**DOI:** 10.3390/ijms22073750

**Published:** 2021-04-03

**Authors:** Jonathan Turpin, Daed El-Safadi, Grégorie Lebeau, Etienne Frumence, Philippe Desprès, Wildriss Viranaïcken, Pascale Krejbich-Trotot

**Affiliations:** PIMIT, Processus Infectieux en Milieu Insulaire Tropical, Université de La Réunion, INSERM UMR 1187, CNRS 9192, IRD 249, Plateforme CYROI, 2, rue Maxime Rivière, 97490 Sainte-Clotilde, Ile de La Réunion, France; jonathan.turpin@univ-reunion.fr (J.T.); daedalsafadi@gmail.com (D.E.-S.); greg.lebeau@live.fr (G.L.); etienne.frumence@univ-reunion.fr (E.F.); philippe.despres@univ-reunion.fr (P.D.)

**Keywords:** Zika virus, ER stress, unfolded protein response, apoptosis, CHOP

## Abstract

Zika virus (ZIKV) is an emerging mosquito-borne flavivirus considered as a threat to human health due to large epidemics and serious clinical outcomes such as microcephaly in new-borns. Like all flaviviruses, ZIKV relies on the cellular machinery to complete its viral cycle, with the endoplasmic reticulum (ER) being the critical site of viral replication factories. The sudden high protein load in the ER induces an ER stress to which the cell responds with an appropriate unfolded protein response (UPR) in an attempt to restore its disturbed homeostasis. When the restoration fails, the cell signalling leads to a programmed cell death by apoptosis with the upregulation of the UPR-induced C/EBP homologous protein (CHOP) which acts as the main trigger for this fatal outcome. Our previous studies have shown the ability of ZIKV to manipulate various cellular responses in order to optimize virus production. ZIKV is able to delay apoptosis to its benefit and although ER stress is induced, the UPR is not complete. Here we discovered that ZIKV impairs the expression of CHOP/*DDIT3*, the main factor responsible of ER-stress driven apoptosis. Surprisingly, the mechanism does not take place at the transcriptional level but at the translational level.

## 1. Introduction

Zika virus (ZIKV) is a flavivirus that belongs to the *Flaviviridae* family including more than 50 distinct species of enveloped viruses, several of which are pathogenic to humans such as Yellow fever virus (YFV), Dengue virus (DENV) and West Nile virus (WNV). In the last ten years, ZIKV almost ignored so far, became a serious threat for human health due to the explosive Brazilian outbreak in 2015 with a number of cases that reached almost one million. During these epidemics, several thousand cases have been recorded with serious neurological complications in adults and congenital malformations like microcephaly in new-borns [1]. This led the World Health Organization to declare ZIKV as a public health emergency of international concern and mobilized the scientific community to address the lack of data on this virus, its interactions with its hosts and its pathogenic mechanisms.

ZIKV is an arbovirus, transmitted by mosquitoes mainly of the *Aedes* genus. ZIKV can also be sexually transmitted which increases the risk of propagation [2]. Like other flaviviruses, ZIKV enters the cells by endocytosis and once its genome is released into the cytoplasm, it is translated to initiate the viral multiplication. Replication of ZIKV-RNA and synthesis of its corresponding viral polyprotein occur in the Endoplasmic Reticulum (ER) invaginations. This results in an ultra-structural modification of the ER with an increased overall size and formation of convoluted membranes [3,4]. It has been reported that ZIKV cycle of multiplication is dependent on several ER proteins including ER membrane complex proteins that are essential for the replication of ZIKV and DENV [5]. The ER is involved in post-translational modifications and folding of secreted and membrane cellular proteins. Like cellular proteins, ZIKV proteins are processed and folded in the ER. Folding requires a constant supply of ATP and a stable concentration of Ca^2+^ necessary for the activity of chaperone proteins like calreticulin, calnexin, Bip/GRP78 and GRP94. These chaperones stabilize protein folding intermediates. They provide a balanced redox environment promoting the formation of disulfide bonds and the tridimensional protein conformation [6].

**Figure 1:**
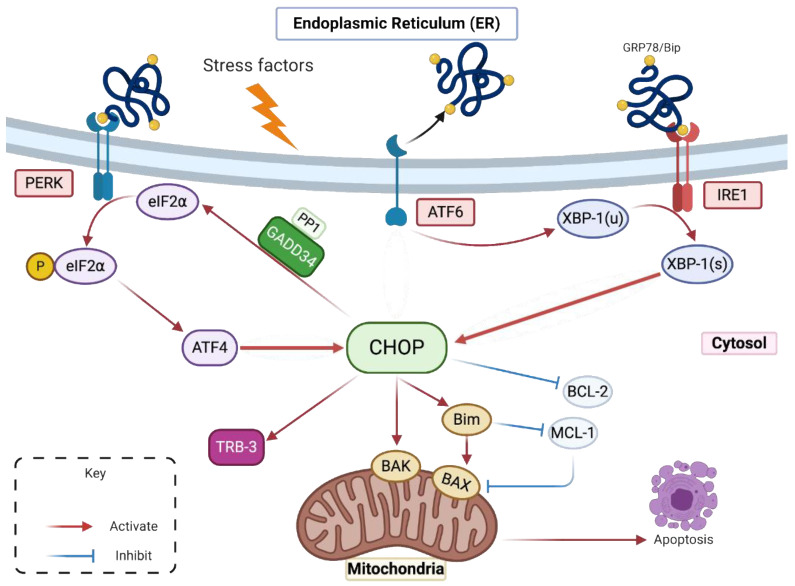
CHOP activation during ER stress and UPR (adapted from [18]).

Multiple conditions are able to disturb the ER homeostasis like glucose starvation, hypoxia, calcium dysregulation or protein accumulation which will in turn induce ER stress [7]. Pharmacological ER stress inducers can mimic these functional defects, such as thapsigargin (TG), a Sarco/endoplasmic reticulum calcium-ATPase pump (SERCA) inhibitor that promotes calcium stock depletion in the ER, Tunicamycin (TM) an inhibitor of N-linked glycosylation and reducing agents like dithiothreitol (DTT) that block disulfide-bond formation and promote protein retention in ER. In response to the ER stress and in order to restore the ER homeostasis, the Unfolded Protein Response (UPR) is activated [8]. Three signalling pathways will help to relieve the cell from ER congestion and stress [9]. The first pathway mediated by the protein kinase R-like endoplasmic reticulum kinase (PERK), is based on attenuating the translation process in order to reduce the influx of newly synthesized proteins in the ER. The second one is the activating transcription factor 6 (ATF6) pathway, which leads to an increase in the ER protein-folding capacity by enhancing the translation of chaperone proteins like Bip/GRP78 and GRP94 [10]. The third pathway depends on the Inositol-Requiring Enzyme 1 (IRE1), favouring the ER-associated degradation (ERAD) process to eliminate the unfolded or misfolded proteins by the ubiquitin-proteasome system [11]. When the stress is prolonged or not properly resolved, cells activate their death program [12,13]. Under these circumstances, the transcription factors (TF): ATF4, ATF6f and the spliced form of the X-box binding protein 1 (Xbp-1(s)), activated respectively by the three UPR branches PERK, ATF6 and IRE1, lead to the upregulation of the C/EBP homologous protein (CHOP) expression. CHOP’s upregulation is the pivotal event that drives to the initiation of cell suicide [14] (Figure 1).

CHOP also known as GADD153 (Growth Arrest and DNA Damage inducible gene 153) and *DDIT3* (DNA-damage-inducible transcript 3), is a transcription factor mainly involved in the ER stress-induced apoptosis. CHOP is known to promote apoptosis in several ways. It induces the upregulation of the Bcl-2 like protein 11 (BIM) which in turn activates the pro-apoptotic mitochondrial factors BAX and BAK [15]. Its overexpression induces the upregulation of several genes promoting cell death by apoptosis and cell cycle arrest, like Tribbles homolog 3 (TRB3), NOXA, p53 upregulated modulator of apoptosis (PUMA) and others [16,17]. Studies have shown that CHOP expression is followed by the suppression of the Bcl-2 anti-apoptotic protein expression through dimerization with c-AMP responsive element binding protein (CREB) [18] but it is still unclear if Bcl-2 expression is directly or indirectly regulated by CHOP.

Under normal conditions, CHOP is ubiquitously expressed at a very low level [18]. Under ER stress and UPR activation, CHOP’s expression increases. Indeed, the transcription of CHOP (*Chop/DDIT3*) encoding gene is under the control of specific UPR inducible TFs mostly ATF4 and Xbp-1(s) [19]. Furthermore, a translational control is also carried out by a phosphorylated alpha subunit of eukaryotic initiation factor (eIF2α) dependent initiation, conditioned by the activated PERK pathway of the UPR system [20]. CHOP is a factor whose expression is highly controlled through transcriptional and translational regulation, probably because of its involvement in cell death.

Many studies have shown that the three branches of the UPR (PERK, ATF6 and IRE1) are activated in cells infected with flaviviruses like DENV, WNV and YFV [21]. Like other flaviviruses, ZIKV infection has been shown to trigger ER stress and UPR activation [22,23,24,25]. For several viruses, the mechanism underlying UPR activation has been extensively explored with a particular focus on identifying the viral determinant that drives it. Thus, it has been shown that the Hepatitis C virus (HCV) Envelope protein is able to promote UPR and control CHOP expression [26]. Concerning DENV, it is reported that Xbp-1 splicing is induced by NS2B/3 [27]. This indicates that specific proteins have the ability to support UPR activation during the flaviviruses cycle of replication. Their action in UPR signalling would thus contribute to the development of a suitable environment for the virus replication, notably through the overexpression of chaperone proteins [28]. However, by promoting several modes of cell defence including antiviral programs, immune responses and involvement in autophagy or apoptosis, the UPR also contributes to thwart the virus [29]. Our team recently reported that ZIKV induces a partial transduction of ER stress by activating only two UPR’s pathways, PERK and IRE1 [25]. We also showed that the virus-induced apoptosis was delayed regarding the virus multiplication cycle [30]. Moreover, we found that ZIKV was not only able to interfere with apoptosis but could even provide protection against an exogenous induction of cell death. This anti-apoptotic activity of ZIKV was shown to be dependent on Bcl-2 protein [30].

As aforementioned, UPR signalling is ultimately deleterious for viral replication. Since CHOP is the main pro-apoptotic transcription factor activated by UPR, we hypothesized that ZIKV might regulate the expression of CHOP protein during the virally induced ER stress. This could be one of the explanations of the delayed apoptosis observed in ZIKV-infected cells.

In a normal state, the chaperone protein GRP78/BiP, the sensor of accumulated or misfolded proteins in the ER, is associated with the luminal domain of three different ER transmembrane proteins to prevent their activation. These three proteins, PERK, ATF6 and IRE1, are stress transducers [12]. In case of ER homeostasis disturbance and during ER stress, GRP78/BiP is dissociated from these proteins activating their stress-signal transducing capacity [31]. (i) Activated PERK phosphorylates eIF2α and leads to a general lowering in mRNA translation and protein influx in the ER [32]. Conversely, phosphorylated eIF2α is required for the translation of ATF4 mRNA. ATF4 protein is a transcription activator of many UPR target genes including *Chop/DDIT3* and DNA damage protein (GADD34) [33]. (ii) Activated ATF6 moves to Golgi apparatus where it is cleaved by site 1 and site 2 proteases generating ATF6f [34]. ATF6f is an activated basic leucine zipper (b-ZIP) factor that reaches the nucleus to promote the transcription of *Chop/DDIT3* and different genes involved in protein re-folding. (iii) IRE1 is a kinase which is auto-phosphorylated in response to ER stress. It is involved in the Xbp-1′(s) unconventional splicing due to its endonuclease activity, leading to a 26 nucleotide-introns removal from Xbp-1 mRNA [35]. Spliced Xbp-1 (Xbp-1-(s)) encodes a b-ZIP transcription factor that contributes to the expression of genes involved in the ERAD and genes involved in protein folding [36]. CHOP triggers apoptosis by inhibiting the Bcl-2 proteins family like BCL-2, MCL-1 and by upregulating Bim, which in turn regulates the mitochondrial outer membrane permeabilization mediated by BAX and BAK. CHOP can induce the upregulation of TRB-3 gene which can also induce apoptosis. In addition, it can directly activate GADD34 that induces the dephosphorylation of eIF2α by combining with protein phosphatase 1 (PP1) [18]. This deregulation with an increase in protein translation and ER stress ends by cell apoptosis.

## 2. Results

### 2.1. Despite an Incomplete UPR, the Factors That Govern the Transcriptional Activation of CHOP Are Present in ZIKV-Infected Cells

The increase of the CHOP gene transcription in response to ER stress may be the result of activation of all three UPR branches and may be due to PERK/ATF4, ATF6 or IRE1/Xbp-1(s) activities (Figure 1). We have recently reported that ZIKV was responsible for inducing an ER stress in A549 cells due to the accumulation of viral proteins [25] and that ZIKV-induced UPR was characterized by PERK and IRE1 activation but not the ATF6 UPR pathway [25]. In order to confirm these results, we measured the expression of UPR-inducible TFs, ATF4 and Xbp-1(s), involved in CHOP expression in ZIKV-infected cells.

**Figure 2:**
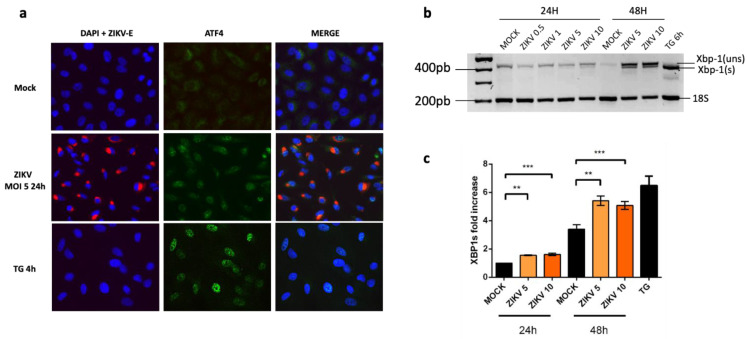
UPR inducible Transcription Factors, ATF4 and Xbp-1(s) are up-regulated in A549 cells infected with ZIKV. (**a**) Cells were infected with ZIKV at MOI = 5 for 24 h or treated with TG for 4 h. Cells were immunostained for ZIKV envelope (ZIKV-E, red) and ATF4 protein (green). Nuclei were stained with DAPI (blue). (**b**) RT-PCR of total Xbp-1 (spliced and unspliced) was performed in A549 cells infected with ZIKV at MOI 1, 5 or 10 for 24 h and 5 and 10 for 48 h post infection. Products were migrated on 2.0% agarose gel and compared to the one obtained in mock-infected cells and in cells treated with TG, the ER stress/UPR pharmacological inducer. (**c**) qRT-PCR analysis of Xbp-1(s) mRNA was performed in A549 cells infected or not with ZIKV at MOI 10 for different times as indicated. Our positive control cells have been treated with TG for 6 h. Data were presented as mean SEM (*n* = 3). *** *p* < 0.001, ** *p* < 0.01, vs. mock.

During UPR, PERK activation and eIF2α phosphorylation are responsible for a general attenuation of the mRNA translation, except specific genes, like ATF4 that escapes this silencing and requires a phosphorylated eIF2α to be translated. We then performed immunofluorescence imaging of ATF4 in order to determine if it was expressed and translocated into the cell nuclei in ZIKV infected A549 cells. ATF4 was clearly detected in nuclei of cells infected 24 h with ZIKV or incubated with ER stress/UPR pharmacological inducer TG as a positive control (Figure 2a).

During UPR, the activated IRE-1 is involved in the unconventional splicing of Xbp-1 with Xbp-1(s) being important for Chop/DDIT3 upregulation. We performed RT-PCR and qRT-PCR to follow the Xpb-1 splicing and mRNA levels in cells infected with ZIKV at several MOI for 24 and 48 h. The results indicate that an Xbp-1 splicing takes place in the infected cells. It depends on the MOI and it is more pronounced at 48 h. (Figure 2b,c).

### 2.2. Upregulation of CHOP Transcriptional Activity in A549 Cells Infected by ZIKV

**Figure 3:**
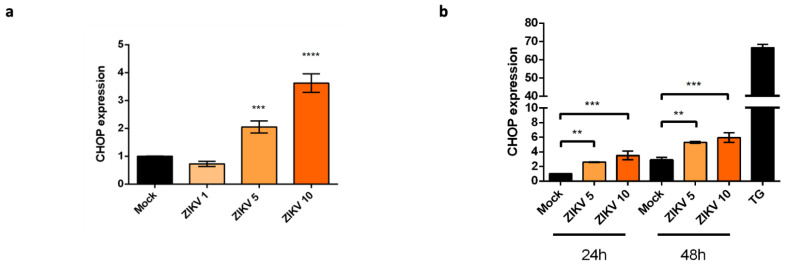
CHOP expression is upregulated at the transcriptional level in ZIKV infected cells. (**a**): qRT-PCR analysis of CHOP mRNA was performed in A549 cells harvested 24 h after being infected with ZIKV at different MOI (1, 5 and 10). CHOP mRNA expression was normalized to the GAPDH mRNA level. Data were presented as mean SEM (*n* = 3). **** *p* < 0.0001, *** *p* < 0.001 vs. mock. (**b**): qRT-PCR analysis of CHOP mRNA was performed in A549 cells harvested at 24 h and 48 h after being infected or not with ZIKV at MOI 5 and MOI 10 or treated with TG for 6 h. CHOP mRNA expression was normalized to the GAPDH mRNA level. Data were presented as mean and SEM (*n* = 3). *** *p* < 0.001, ** *p* < 0.01 vs. mock.

The nuclear localization of ATF4 and the splicing of Xbp-1 suggest that these UPR-induced TFs may be sufficient to upregulate *Chop/DDIT3* transcription in ZIKV infected cells. We performed qRT-PCR analysis of CHOP mRNA. We found a 2- and 4-fold increase in ZIKV-infected cells at MOI 5 and MOI 10 respectively, compared to the uninfected cells (Figure 3a). If we follow the evolution of this upregulation over time, we can see that the increase in CHOP mRNA levels is significant 24 and 48 h post-infection, although modest compared to that obtained with the ER stress/UPR pharmacological inducer TG (Figure 3b). The amounts of CHOP mRNA detected depend on the MOI and they increase during the time course of infection. We conclude that, although ZIKV triggers an incomplete UPR, the infection still leads to an upregulation of CHOP at the transcriptional level.

### 2.3. ZIKV Inhibits CHOP Protein Expression

**Figure 4:**
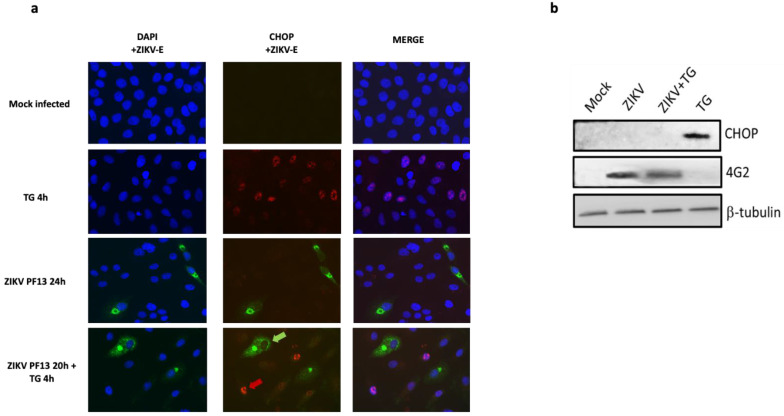
ZIKV inhibits CHOP protein expression in the infected cells. (**a**) Immunofluorescence detection of ZIKV envelope (ZIKV-E, green) expressing cells and CHOP protein (red) in A549 cells infected or not with ZIKV at MOI = 5 for 24 h, further treated or not with TG for 4 h at 20 h.p.i and cells treated with TG for 4 h. Nuclei were stained with DAPI (blue). h.p.i: hours post-infection. In the microscopic field of cells infected with ZIKV and treated with TG for 4 h at 20 h.p.i, the green arrow points a cell stained for ZIKV-E without CHOP staining in the nucleus and the red arrow points a cell which is not stained for ZIKV-E but has a CHOP stain in the nucleus. (**b**) A549 cells were infected or not with ZIKV at MOI 5 for 24 h, treated 20 h.p.i with TG for 4 h or only treated with TG for 4 h. The cells were harvested at the different conditions indicated above and subjected to western blot analysis using antibodies against CHOP and ZIKV envelope protein (4 G2). β-Tubulin was used as a loading control.

To gain insight into the regulation of CHOP protein expression and its localization during ZIKV infection, we used immunofluorescence imaging. A549 cells were infected with ZIKV at MOI 5 for 24 h. Stressed cells, treated with TG for 4 h, were used as an UPR positive control regarding upregulation of CHOP synthesis. ZIKV-E detection allowed the monitoring of the infected cells 24 h after ZIKV addition at MOI 5. Unlike TG treated cells, which showed a strong CHOP signal in their nuclei, no CHOP protein could be detected in the nucleus of ZIKV infected cells (Figure 4a). Despite the ER stress and UPR induction triggered by ZIKV as previously shown and despite a transcriptional activation of CHOP gene, CHOP protein seems to be lacking in the infected cells.

To understand whether ZIKV was able to control the expression or the localization of CHOP protein, an exogenous UPR inducer (TG) was added to the infected cells 20 h post infection for a treatment of 4 h. Again, CHOP signal was undetectable in the infected cells, immunostained for ZIKV-E. In contrast, in the same microscopic fields, uninfected cells without any ZIKV-E immunostaining, do express CHOP protein with a staining being clearly nuclear (Figure 4a). These observations suggest that ZIKV infected cells lack a nuclear CHOP protein, despite an exogenous induction of UPR by TG addition 20 h after the virus.

We further performed Western Blotting (WB) analysis to compare CHOP protein patterns in the different conditions. A technical adaptation of the classical WB with a glutaraldehyde prefixation step of the blotted membranes was necessary to allow a clear immunodetection of CHOP. This step improves the retention of low molecular weight acidic proteins such as CHOP on the membrane and we followed exactly the same steps as mentioned [37]. Firstly, the immunoblot confirmed that CHOP protein was undetectable in the control cells that are uninfected and unstressed. This was not surprising and has already been documented [37]. Indeed, despite a basal level of transcription (Figure 3), CHOP protein is not produced in cells in normal conditions, due to a lack of translation of the transcripts. Secondly, as previously revealed by immunofluorescence, CHOP protein was detectable in extracts from TG-treated cells, indicating that its expression was induced under ER stress conditions. Finally, the WB analysis confirmed that CHOP was undetectable in ZIKV infected cells, whether they have been treated additionally with TG or not (Figure 4b).

We wondered whether the lack of CHOP detection was related to a direct effect of ZIKV infection on the conformation of the protein, resulting in a loss of antigenic reactivity. To rule out this hypothesis, we performed immunofluorescence using several anti-CHOP antibodies including a polyclonal one. We obtained the same images with CHOP missing in the infected cells in contrast to the TG treated cells (Supplementary Materials, Figure S1).

We then examined whether a lack of CHOP expression was a characteristic of the cell line used and/or a specificity associated with the viral strain of ZIKV. We therefore replicated the experiment with HuH7 cell line and with the ZIKV-MR766 molecular clone, corresponding to the historical African lineage of the virus. The same observations were achieved on HuH7 and with ZIKV-MR766 (Figure S2).

In addition to the exogenous stress inducer, we also added a proteasome inhibitor, as we could not exclude the possibility that CHOP was synthesised and rapidly degraded in the infected cells, thus explaining the inability to detect it. Again, we were able to verify that even in the presence of this inhibitor and while CHOP was detectable and localised in the nuclei of uninfected cells, it was not detectable in cells with ZIKV-E labelling (Figure S2).

Together, these results support the hypothesis that ZIKV, although inducing ER stress, UPR and upregulation of CHOP transcription, inhibits the production of the CHOP protein. Moreover, ZIKV has the capacity to prevent CHOP protein expression in infected cells even in the presence of the UPR inducer TG.

### 2.4. ZIKV Controls the Transcriptional Activity of CHOP-Dependent Genes

To ensure that the lack of CHOP observation in infected cells was not due to a loss of sensitivity in the detection system, we investigated whether we could have an evidence of a biological activity that could attest for the presence of CHOP, even though we could not detect it.

As a transcription factor, CHOP regulates the expression of several pro-apoptotic genes, including TRB-3, BIM, PUMA and NOXA as previously mentioned [16,17]. We checked the transcriptional levels of these genes in different conditions: ER stress/UPR pharmacological induction with TG, ZIKV infection or both.

**Figure 5:**
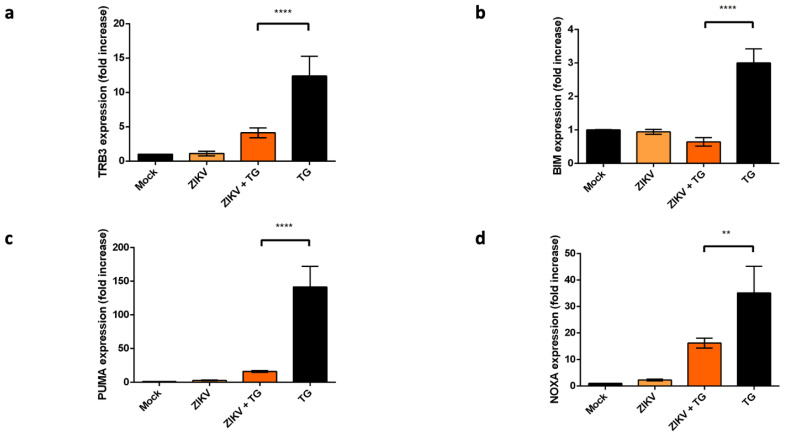
Transcriptional activity of CHOP-dependent genes. qRT-PCR analysis of TRB3 (**a**), BIM (**b**), PUMA (**c**) and NOXA (**d**) mRNAs were performed in A549 cells. The cells were harvested 24 h after being or not infected with ZIKV at MOI = 5 or treated 4 h with TG 20 h.p.i with ZIKV. Our positive control cells were treated with TG for 4 h. Data were presented as mean SEM (*n* = 3). **** *p* < 0.0001, ** *p* < 0.01 vs. mock.

Compared to the mock, the expression of TRB-3, BIM, PUMA and NOXA mRNA showed a slight to no increase in ZIKV infected cells (Figure 5a–d). The increase factors are not comparable to those obtained in UPR conditions induced by the TG treatment, which is associated with a nuclear detection of CHOP protein.

In A549 cells infected 20 h with ZIKV, TG was poorly efficient for inducing transcriptional activity of the CHOP-stimulating genes TRB3, BIM, NOXA and PUMA as compared to mock, infected cells. A such result suggests that ZIKV has ability to prevent the ER stress-mediated activation of pro-apoptotic genes in relation with CHOP factor.

## 3. Discussion

The ER is an intracellular compartment that plays a major role in cell biological activities. More than a third of the total proteins produced in the cells, especially those destined for residence in ER, Golgi apparatus, lysosomes or cytoplasmic membrane are synthesized, folded and processed in the ER [38]. Several factors can break the ER homeostasis, trigger ER stress and further induce an accumulation of misfolded or unfolded proteins. In order to cope with the ER stress, the cells activate UPR. This response is in principle cytoprotective, reestablish the ER features and prevent the cytotoxic effect of the unfolded accumulated proteins. If the ER stress is severe, prolonged and not properly resolved, the UPR ends up with cellular apoptosis [39]. The C/EBP homologous protein CHOP is a transcription factor induced by UPR system and considered as a pivotal trigger for ER stress-induced apoptosis. A convergence and persistence of UPR pathways govern the expression of CHOP, as Chop/DDIT3 transcriptional activation is known to be under the control of the stress transcription factors ATF4, ATF6f and Xbp-1(s). Several studies have shown that a partial UPR activation does not affect CHOP expression with PERK/ATF4 and IRE1 pathways remaining the main pathways responsible for the induction of CHOP expression [40]. CHOP expression is also conditioned by the shift between unphosphorylated and phosphorylated forms of eIF2α during UPR, shift also required for ATF4 translation [33]. Once expressed and translocated in the nucleus, CHOP is a transcriptional activator of several genes among which are pro-apoptotic genes (Figure 1). Apoptosis that follows CHOP upregulation is thus due to CHOP’s ability to tip the balance between anti- and pro-apoptotic factors towards a dominant pro-apoptotic activity.

During their replication, flaviviruses induce ER remodelling with membrane proliferation and ER lumen expansion related to accumulation of viral structural and non-structural proteins [41]. An excessive folding activity is then prone to induce an ER stress in the infected cells and lead to apoptosis in the face of an impossible return to normality. The activation of the apoptosis promoting transcription factor CHOP during UPR is then emerging as a general response to ER-tropic viruses, replicating in the ER [42,43].

Given that a relative cell health is the guarantee that drives efficient viral multiplication and that, symmetrically, UPR and apoptosis can be effective antiviral responses, it is not surprising that viruses interfere with the crosstalk between ER stress, UPR and apoptosis responses. The flavivirus family is no exception to the rule. Several, like DENV and WNV have been shown to activate the three UPR branches [44,45]. Other modulates the host UPR signalling pathways in order to promote viral replication and maintain their persistence in infected cells [28]. Our recent study has shown that only the PERK and IRE1 branches are activated by ZIKV with the ATF6 branch that seems to be inhibited in A549 infected cells [25]. Concerning the issues related to the ER stress outcome, many viruses have shown their capacity to trigger an UPR that upregulates the expression of CHOP protein and leads to cell death. HCV activates UPR and induces apoptosis by upregulating GADD153/CHOP expression [46]. Both WNV and Japanese encephalitis virus (JEV) elicit UPR and initiate apoptosis by the induction of CHOP [43,47]. On the other hand, other studies showed that some viruses do not induce the expression of CHOP protein despite their induction of ER stress and activation of the UPR system. One of these viruses is the murine coronavirus, mouse hepatitis virus (MHV) that activates only a part of the PERK UPR branch with ATF4 expression but without its downstream genes GADD153/CHOP and GADD34 [46].

Given that ZIKV induces ER stress but leads to incomplete UPR [25], we focused on the expression of CHOP in order to understand the mechanism that delays apoptosis in ZIKV-infected A549 cells [30]. We found that although *Chop/DDIT3* transcription was upregulated (Figure 3), we could not detect the CHOP protein in ZIKV infected cells (Figure 4). This was coherent with an UPR leading to increasing amounts of spliced Xbp-1 and nuclear translocation of ATF4 (Figure 2). As CHOP transcripts were detectable, a non-detectable CHOP at the protein level raised several questions. CHOP could have been expressed in the presence of ZIKV but at a level below the threshold of immunodetection. A quantitative check by Western Blotting, after a fixation step of the transferred proteins on the NC membrane [37] confirmed the lack of CHOP in infected cells. More surprisingly, ZIKV was able to induce a missing CHOP despite addition of an exogenous UPR inducer. Analysis of the transcripts of pro-apoptotic genes, whose expression is ‘CHOP-dependent’, like TRB3, BIM, PUMA and NOXA, shows no activation of their transcription. The lack of CHOP target genes upregulation reinforces the fact that CHOP protein is absent. In addition, a defect in the CHOP protein may also explain why we previously found that ZIKV-infected cells exhibited an increased amount of the anti-apoptotic protein Bcl-2. The upkeep of Bcl-2, normally down-regulated by CHOP can be a significant advantage in maintaining an anti-apoptotic environment during the time of viral replication [30].

The total lack of a CHOP protein signal in ZIKV infected cells or in cells infected and treated with a pharmacological inducer of UPR does not support the hypothesis that CHOP protein synthesis took place and was followed by its rapid degradation. The most likely reason for the absence of CHOP is a lack of protein synthesis due to a translational defect. Therefore, we hypothesize that ZIKV interferes with a complete UPR activation [25] to ensure persistent ER stress which will be useful to the virus to increase its replication. This leads to CHOP transcriptional activation, but to escape the pro-apoptotic effects of CHOP protein, ZIKV is able to inhibit the translation of CHOP messengers.

The translation of CHOP is known to be dependent on phosphorylated eIF2α. Since we have shown that the PERK pathway is active in ZIKV infected cells, with an effective production of the ATF4 factor (under the same translational control), the lack of CHOP translation cannot be attributed to an eIF2α phosphorylation defect. The way in which ZIKV leads to an inability of CHOP mRNAs to be translated requires further investigation. It will be interesting to identify which specific ZIKV determinants are involved in UPR induction and CHOP regulation. To decipher which viral determinant is able to interfere with CHOP expression, experiments in which each viral protein could be individually expressed need to be conducted. If ZIKV interferes with UPR through the binding of a viral factor to a host protein, identification of the partners could further be achieved using mass spectrometry or nuclear magnetic resonance, following co-immunoprecipitation. This will provide insights into the mechanistic details of ER stress-mediated cell survival and apoptosis during ZIKV infection. Interestingly, it will make it possible to identify how to disrupt a key mechanism of the cell suicide in situations of persistent and pathological ER stress. It is of note that some research has been conducted on other viruses and might guide our future work on such a topic. Depending on the virus, structural or non-structural proteins have been identified as being able to act on the UPR pathways leading to apoptosis. The capsid protein and its import into the cell nucleus have been shown to be a major player in the control of these cellular responses [48,49,50,51]. A viral non-structural protein NSP2-mediated mechanism has been reported for Chikungunya virus ability to shut-off UPR pathways [52]. In addition, American Swine Fever virus was shown to inhibit CHOP induction through recruitment of protein phosphatase 1 by its DP71L protein, leading to dephosphorylation of eIF2α [53]. Concerning the Cytomegalovirus, a M50-dependent proteasomal degradation of IRE1 has been observed, inhibiting the UPR fatal outcomes in infected cells [54]. Surprisingly, such interaction between ZIKV viral factors and UPR-related proteins remains poorly documented, except recently for E protein and GRP78/BiP [55], and represents a crucial scope for future investigations.

It might also be interesting to study how ZIKV deals with the presence of a functional CHOP protein, as there are no studies that establish the effect of CHOP on viral replication. Infectious Bronchitis Virus (IBV)-induced apoptosis is attenuated in CHOP-deficient cells and virus replication is inhibited [56]. In contrast, in CHOP-deficient mouse embryonic fibroblasts (MEFs), WNV infection increases significantly compared to wild-type MEFs [44]. These studies suggest that CHOP-induced apoptosis is beneficial to IBV replication but not to WNV replication. It would be interesting to know what is going on with ZIKV.

## 4. Materials and Methods

### 4.1. Virus, Cell Culture, Antibodies and Reagents

For ZIKV we used the clinical isolate PF13 (French Polynesia, 2013) which is the epidemic strain of Asian origin [57] and a molecular clone of MR766 which is the historical strain of African origin [58]. A549 cells (ATCC, CCL-185) and HuH7 were cultured at 37 °C under a 5% CO2 atmosphere in MEM medium supplemented with 10% of heat-inactivated foetal bovine serum (FBS).

Immunodetection of the viral envelope protein was performed using either the rabbit anti-EDIII ZIKV or the mouse anti-pan flavivirus envelope E protein monoclonal antibody (4G2), produced by RD Biotech. To detect ATF4 and CHOP proteins expression, we used the monoclonal rabbit anti-ATF4 and the mouse monoclonal anti-CHOP, 9C8 (in Figure 4) from ThermoFisher and the rabbit polyclonal anti-CHOP/GAD153 from BioVision (Figure S1). For the secondary antibodies, donkey anti-mouse Alexa Fluor 488 and anti-rabbit Alexa Fluor 594 IgG antibodies were obtained from Invitrogen (ThermoFisher, Les Ulis, France). To induce ER stress and activate the UPR, Thapsigargin (TG), an endoplasmic reticulum Ca^2+^-ATPase inhibitor was used at 1 μM for an incubation time of 4 h. TG was purchased from Sigma-aldrich (Humeau, La Chapelle-Sur-Erdre, France). To induce proteasome inhibition, we used bortezomib/Velcade purchased from Janssen-Cilag, Millennium Pharmaceuticals, Inc., Cambridge, MA USA, and Johnson & Johnson Pharmaceutical Research & Development, L.L.C., Raritan, NJ, USA.

### 4.2. RNA Extraction and qRT-PCR

Total RNA has been extracted from cells by using the RNeasy Plus Mini Kit (Qiagen, Hilden, GERMANY cat. nos. 74134 and 74136). The total cDNA was obtained by RT using E reverse primer (5′-TTCACCTTGTGTTGGGC-3′) and M-MLV reverse transcriptase enzyme at 42 °C for 50 min. cDNA was subjected to a quantitative PCR, using a CFX96 Real Time Detection System (BioRad, Hercule, CA, USA). For amplification, GoTaq Master Mix (Promega, Charbonnières-les-bains, France) and different specific primers were used to follow ATF4, Xbp-1(s), GRP78, CHOP, TRB3, BIM, PUMA, NOXA and GAPDH gene transcripts. A threshold cycle (Ct) was calculated for each single sample amplification reaction using the CFX96 program (Bio-Rad) in the exponential phase of amplification. CHOP was normalized to the glyceraldehyde-3-phosphate dehydrogenase (GAPDH) reference.

### 4.3. Immunofluorescence Assay

A549 cells were grown, infected with ZIKV at MOI 5 or treated with TG on glass coverslips. They were further fixed with 3.7% paraformaldehyde at room temperature for 10 min. Fixed cells were permeabilized with 0.1% Triton X-100 in PBS for 5 min. Coverslips were incubated with primary antibodies (1:1000 dilution) in 1% BSA-PBS 1X for two hours to block nonspecific binding of the antibodies and then with Alexa Fluor-conjugated (Alexa 488 and Alexa 594) secondary antibodies (1:1000, Invitrogen) for one hour. 4′,6-Diamidino-2-phenylindole Dihydrochloride (DAPI) staining (1:500) was used to reveal the nucleus morphology. Cells were washed 3 times with PBS for 5 min between each incubation. VECTASHIELD^®^ purchased from Vector Lab, Eurobio scientific, Les Ulis, France, was used to mount the coverslips. Slides were kept at 4 °C and fluorescence was then visualized using a Nikon Eclipse E2000-U microscope. By using a Hamamatsu ORCA2 ER camera and the imaging software NIS-Element AR, images were captured and processed.

### 4.4. Cell Extracts Preparation and Western Blotting Optimization

Cells were infected with ZIKV at MOI 5, treated only with TG or treated with TG post-infection and harvested at the indicated time points as presented in the legend of the Figure 3 and Figure 4. To lyse the cells, they were rapidly washed before with Phosphate buffer saline (PBS) and lysed at the concentration of 1 × 10^4^ cells.µL^−1^ with the RIPA (radioimmunoprecipitation assay) lysis buffer added drop by drop while keeping the tubes containing the cells on ice. The lysates were then collected in tubes, sonicated and centrifuged for 20 min at 12,000 rpm. The supernatant was collected and treated with BCA for the protein dosage. These fractions were next used for western blots. The proteins were separated by 10% SDS-PAGE and transferred onto nitrocellulose membrane.

In order to improve the detection of CHOP protein, an additional glutaraldehyde (GA) fixing step has been added to the conventional protocol as previously indicated [37]. The nitrocellulose membrane was fixed with glutaraldehyde (GA) 0.5% in PBS 1X for 5 min. The membrane was next washed three times with PBS–Tween (0.1%) and blocked using 5% non-fat dry milk in PBS-Tween for 1 h at room temperature. Then it was incubated with the mouse anti-CHOP antibody (1:1000 dilutions in PBS-Tween) over night (ON) at 4 °C. Anti-mouse immunoglobulin-horseradish peroxidase conjugated was used as secondary antibody (1:2000 dilutions in PBS-Tween). The membrane was washed three times for 5 min each with PBS-Tween and then incubated for 1 h with the secondary antibody. The blots were detected using chemiluminescence detection kit (Amersham ECL Select) and exposed on an Amersham imager 680.

### 4.5. Statistical Analysis

Our results were statistically analyzed using the software Graph-Pad Prism 7.0. One-way ANOVA test and Student *t*-test were performed to compare between the different experimental conditions. Values of *p* < 0.05 were considered statistically significant for Student *t*-test. The degrees of significance are indicated on the figure. *** *p* < 0.001, ** *p* < 0.01, * *p* < 0.05.

## 5. Conclusions

**Figure 6:**
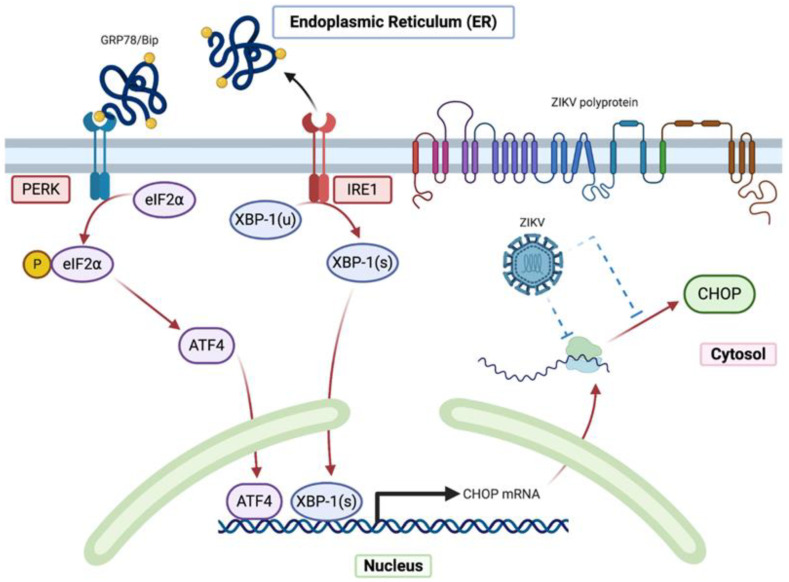
Graphical overview of the impaired expression of CHOP during ZIKV infection.

Together, the results allowed us to conclude that ZIKV, even though responsible for ER stress and associated UPR, impaired the expression of CHOP and consequently the upregulation of its proapoptotic targets. Surprisingly, ZIKV did not affect the expected upregulation of CHOP mRNA transcripts in the A549 infected cells but prevented the CHOP protein expression, also in presence of an exogenous UPR inducer. Ultimately, our research reveals that ZIKV regulates CHOP expression at either translational or post-translational level (Figure 6).

## Data Availability

The data presented in this study are available in this paper and Supplementary Materials.

## Supplementary Materials

The following are available online at https://www.mdpi.com/article/10.3390/ijms22073750/s1, Figure S1: Immunofluorescence detection of ZIKV envelope (ZIKV-E,DIII, green) expressing cells and CHOP protein (red) in HuH7 cells infected or not with ZIKV-MR766 at MOI = 5 for 24 h, further treated or not with TG and treated or not with Bortezomib for 4 h at 20 h.p.i. CHOP nuclear localization was controlled with the panel of cells treated with TG for 4h. Nuclei were stained with DAPI (blue). h.p.i: hours post-infection, Figure S2: Immunofluorescence detection of ZIKV envelope (ZIKV-E,DIII, green) expressing cells and CHOP protein (red) in HuH7 cells infected or not with ZIKV-MR766 at MOI = 5 for 24 h, further treated or not with TG and treated or not with Bortezomib for 4 h at 20 h.p.i. CHOP nuclear localization was controlled with the panel of cells treated with TG for 4 h. Nuclei were stained with DAPI (blue). h.p.i: hours post-infection.
