# Supplementary material for: CHOP Pro-Apoptotic Transcriptional Program in Response to ER Stress Is Hacked by Zika Virus
Source: Int J Mol Sci. 2021 Apr 3;22(7):3750. doi: 10.3390/ijms22073750 (PMC8038490; doi:10.3390/ijms22073750)
Supplement: Supplementary file 1 [file ijms-22-03750-s001.pdf]

Supplementary Materials

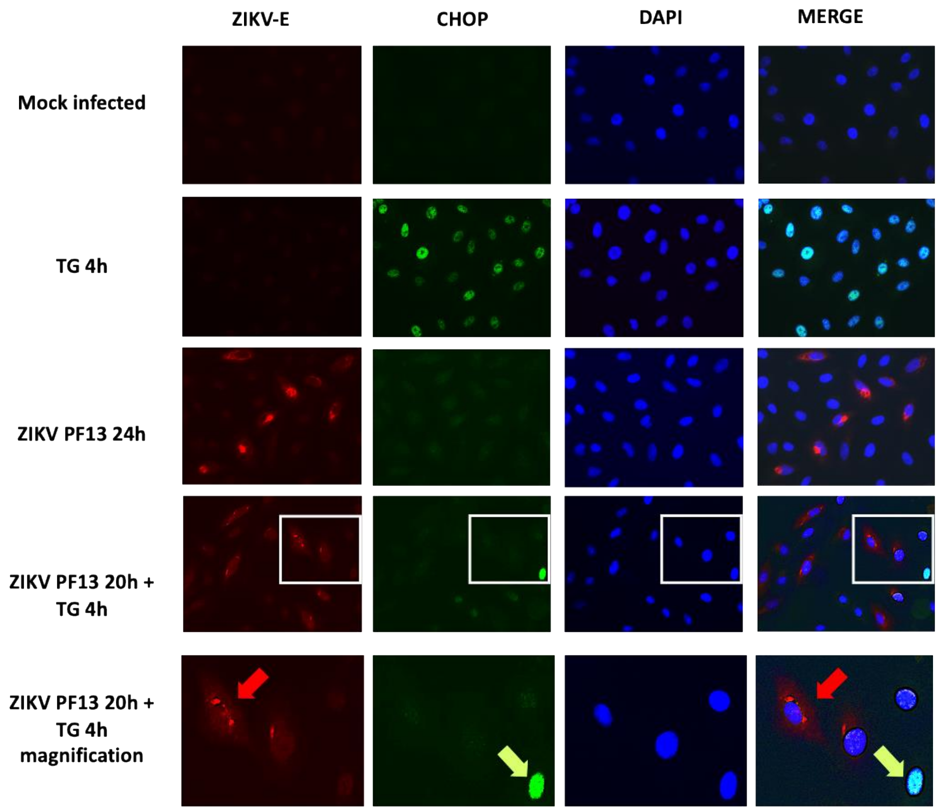

Figure S1.

Immunofluorescence detection of ZIKV envelope (ZIKV-E, 4G2 antibody, red) expressing cells and CHOP protein (green) in A549 cells infected or not with ZIKV at MOI=5 for 24h, further treated or not with TG for 4h at 20 h.p.i and cells treated with TG for 4h. The anti-CHOP used was the polyclonal anti CHOP/GAD153 from BioVision. Nuclei were stained with DAPI (blue). h.p.i: hours post-infection. A square magnification of the microscopic field of cells infected with ZIKV and treated with TG for 4h at 20 h.p.i, indicate with the red arrow a cell stained for ZIKV-E without CHOP staining in the nucleus and with the green arrow a cell which is not stained for ZIKV-E but has a CHOP staining in the nucleus.

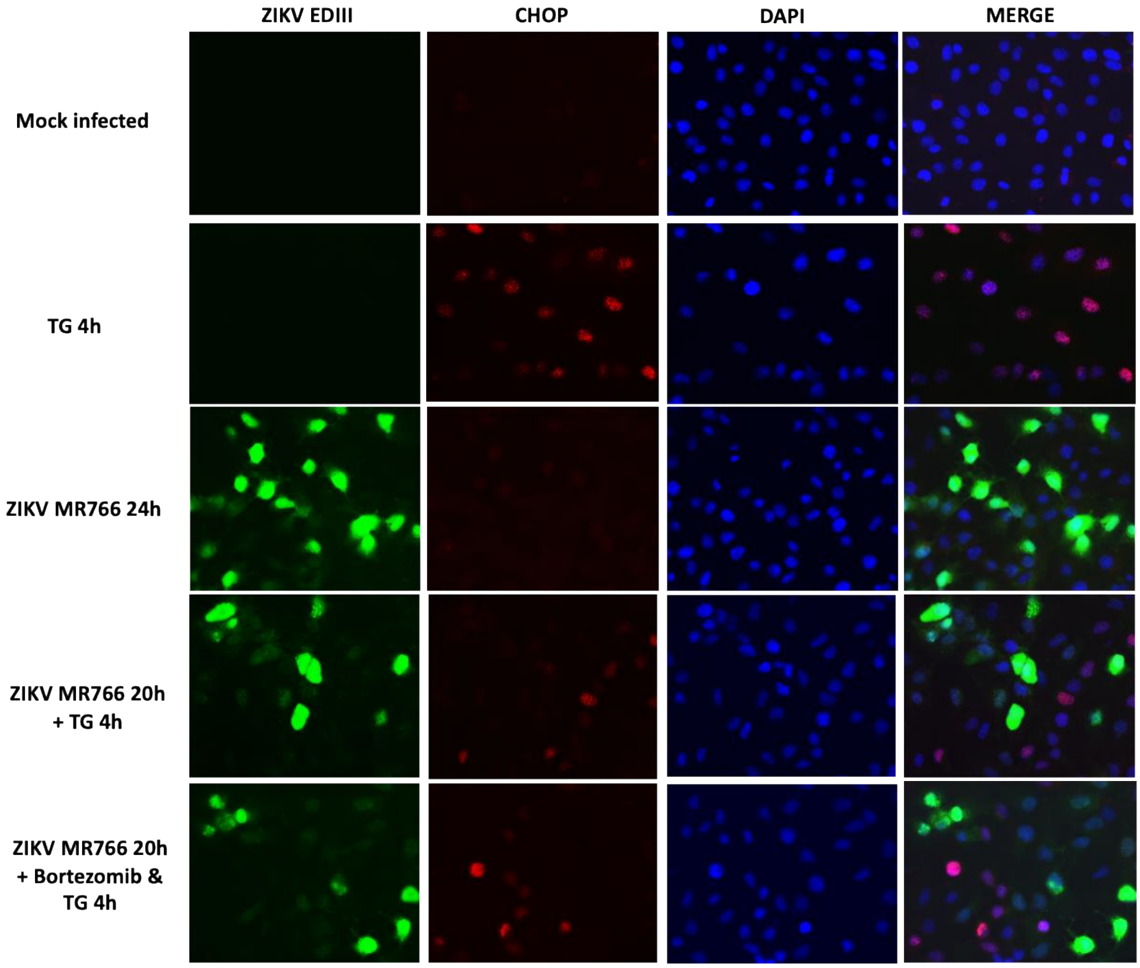

Figure S2.
Immunofluorescence detection of ZIKV envelope (ZIKV-E,DIII, green) expressing cells and CHOP protein (red) in HuH7 cells infected or not with ZIKV-MR766 at MOI=5 for 24h, further treated or not with TG and treated or not with Bortezomib for 4h at 20 h.p.i. CHOP nuclear localization was controlled with the panel of cells treated with TG for 4h. Nuclei were stained with DAPI (blue). h.p.i: hours post-infection.
